# Chitosan Coating Functionalized with Flaxseed Oil and Green Tea Extract as a Bio-Based Solution for Beef Preservation

**DOI:** 10.3390/foods12071447

**Published:** 2023-03-29

**Authors:** Cíntia G. Mendes, Joana T. Martins, Fernanda L. Lüdtke, Ana Geraldo, Alfredo Pereira, António A. Vicente, Jorge M. Vieira

**Affiliations:** 1 CEB—Centre of Biological Engineering, University of Minho, 4710-057 Braga, Portugalfludtke@ceb.uminho.pt (F.L.L.); avicente@deb.uminho.pt (A.A.V.); d8778@ceb.uminho.pt (J.M.V.); 2 LABBELS—Associate Laboratory, 4710-057 Braga, Portugal; 3 Mediterranean Institute for Agriculture, Environment and Development (MED), Institute for Advanced Studies and Research, University of Evora, 7006-554, Évora, Portugal; ana.geraldo@montedopasto.pt (A.G.); apereira@uevora.pt (A.P.)

**Keywords:** packaging, meat, essential oils, textural parameters, lipid oxidation, shelf-life analysis

## Abstract

Ecological and safe packaging solutions arise as pivotal points in the development of an integrated system for sustainable meat production. The aim of this study was to assess the effect of a combined chitosan (Ch) + green tea extract (GTE) + essential oil (thyme oil, TO; flaxseed oil, FO; or oregano oil, OO) coating on the safety and quality of vacuum-packaged beef during storage at 4 °C. An optimized bio-based coating formulation was selected (2% Ch + 2% GTE + 0.1% FO) to be applied to three fresh beef cuts (shoulder, Sh; knuckle, Kn; Striploin, St) based on its pH (5.8 ± 0.1), contact angle (22.3 ± 0.4°) and rheological parameters (viscosity = 0.05 Pa.s at shear rate > 20 s^−1^). Shelf-life analysis showed that the Ch–GTE–FO coating delayed lipid oxidation and reduced total viable counts (TVC) and *Enterobacteriaceae* growth compared with uncoated beef samples over five days. In addition, Ch–GTE–FO coating decreased total color changes of beef samples (e.g., ∆E* = 9.84 and 3.94, for non-coated and coated Kn samples, respectively) for up to five days. The original textural parameters (hardness, adhesiveness and springiness) of beef cuts were maintained during storage when Ch–GTE–FO coating was applied. Based on the physicochemical and microbial characterization results, the combination of the Ch–GTE–FO coating developed was effective in preserving the quality of fresh beef cuts during refrigerated storage along with vacuum packaging.

## 1. Introduction

Food products of animal origin, such as meat and meat derivatives, are a highly valuable source of nutrients (e.g., proteins and essential amino acids) for the human diet. However, their perishability requires proper processing and handling to extend their shelf life. Meat and its derivatives are susceptible to chemical and microbiological spoilage, representing a high health risk to the consumer and economic losses to the food industry [1]. The chemical deterioration of meat is affected by factors such as composition, air, light and processing temperature, which lead to oxidation reactions, namely lipid oxidation [2]. Oxidative damage occurs due to the uncontrolled generation of free radicals and reactive species, leading to quality decay in terms of flavor, color, texture and nutritional value [3,4,5].

Beef cuts, such as striploin (St), shoulder (Sh) and knuckle (Kn), are usually preserved in polyvinyl chloride (PVC) packaging and/or in vacuum packaging [6,7]. However, vacuum packaging causes a change in the color of the meat to a purple-red tone associated with the formation of deoxymyoglobin. On the other hand, PVC packaging allows the beef to be exposed to oxygen, favoring the oxidation of myoglobin. This oxidative reaction leads to an accumulation of metmyoglobin in the meat and a discoloration from a red to a brown color [7]. In both cases, the beef is exposed to oxidative reactions, resulting in a short shelf life [8,9]. In addition, pathogens found in food, such as pathogenic bacteria, tend to cause spoilage in meat by forming biofilms on its surface, which are also responsible for physical, chemical and sensory changes [4,10,11].

Edible coatings present an interesting approach to preserving and packaging meat. They can be applied by spraying, spreading or dipping, forming a thin transparent layer on the food surface [12]. Usually, edible coatings are composed of biopolymers, generated from food industry waste or underutilized sources of proteins, lipids or polysaccharides [13]. In addition, they have the advantage of being biodegradable and edible, and they can load functional agents from natural sources, such as vegetable oils, waxes, natural resins, essential oils, extracts, emulsifiers and surface-active agents to improve the meat products’ shelf life [3,4,14]. In fresh and processed meat, the incorporation of lipids in the coating can improve hydrophobicity and cohesion, forming excellent moisture barriers. Thus, in addition to preventing moisture loss, coatings also retard microbial spoilage by restricting the growth of pathogenic microorganisms and slowing down the oxidation of lipids, proteins and pigments [13]. The interest in the use of edible coatings comes as a response to environmental concerns such as the increase of plastic waste, the need to reduce food industrial waste and consumer demands for more natural and healthy foods [3,4,15].

Polysaccharides, such as chitosan (Ch), are widely used for the development of a variety of food coatings. Ch is a functional biopolymer obtained through the deacetylation of chitin, showing effective inhibition of bacterial, yeast and fungal growth [16]. Natural antioxidants derived from plants, fruits, animals or their by-products have been studied and incorporated into bio-based coatings as these sources are rich in phenolic compounds with antioxidant capacity [4]. Green tea is a valuable source of phenolic compounds, being reported as a scavenger of free radicals that promotes lipid oxidation in food systems, and it shows in vitro inhibitory effects against food spoilage and pathogenic microorganisms [16,17]. In addition, essential oils from plants such as oregano essential oil (OO) and thyme essential oil (TO), which contain thymol and carvacrol, show strong antioxidant and antibacterial properties against a wide range of food pathogens [18,19]. One of the challenges of using essential oils as natural additives is how to overcome the high extraction cost and the influence of their aroma/flavor on coated food [9]. To circumvent this issue, an unexplored alternative could be the use of oil extracted from flaxseed, whose gum has been effectively used as a functional agent in edible coatings, preserving the sensory qualities and safety of food products. In addition, flaxseed oil (FO) presents antioxidant, anti-inflammatory and anti-cancer properties and has no intense odor [20].

Therefore, in this context, different essential oils (i.e., OO, TO and FO) were individually incorporated into a Ch–green tea extract (GTE) solution to formulate novel and effective bio-based coatings to be applied on fresh meat. A Central Composite Rotational Design (CCRD) was performed with the aim to select an optimized Ch–GTE–essential oil coating formulation to be applied onto the surface of different beef cuts (Sh, Kn and St). The selected bio-based coating capacity to preserve the quality of stored vacuum-packed beef cuts was assessed over 8 days under refrigerated storage conditions.

## 2. Materials and Methods

### 2.1. Materials

Ch (>90% deacetylation) was obtained from Golden-Shell Co. China and L(+)-Lactic acid (90%) was obtained from Acros Organics (Belgium). Organic virgin FO—Nature foods (nutritional value per 100 g: 11 g saturated, 18 g monounsaturated and 71 g polyunsaturated fatty acids); OO (*Origanum compactum*)—Biover; and TO—Integralia were all purchased from Celeiro (Dietimport S.A., Portugal). Di-terc-butyl-methyl phenol (BHT), trichloroacetic acid (TCA) and 2-thiobarbituric acid reagent (TBA) were purchased from Merck (Germany).

### 2.2. Beef Samples

The beef samples were obtained from cattle carefully treated under strict sanitary protocol and optimized nutritional feed conditions by Monte do Pasto Lda (Portugal) from calf status until they reached an ideal weight (approx. 448 kg) and age (average: 16 months) for slaughter. All animals (*n* = 15 females) were slaughtered on the same day. During the cutting process, different cut samples were taken from the St (*longissimus dorsi* muscle), chuck Sh clod (composed of *musculus trapezius*, *musculus deltoideus*, *musculus supraspinatus* and *musculus infraspinatus*) and Kn (*quadriceps femoris* muscle) in order to assess the behavior of support muscle (St) and locomotion muscles (Sh and Kn) under coating and non-coating conditions. Samples were analyzed in situ before application of the coating to characterize beef samples at time 0 d (i.e., 48 h post-mortem). The vacuum packaging and the coating application are explained in detail in Section 2.5.

### 2.3. Experimental Design and Coating Preparation

A 2^3^ CCRD with three replicates at the central point and six axial point essays was performed. Ch and essential oil (i.e., TO, FO or OO) concentrations were chosen based on a preliminary experimental design (Plackett–Burman design; Table S1). Ch (0.19–2%, *w*/*v*) and essential oil (0.01–0.12%, *w*/*v*) concentrations were chosen as independent variables. The importance of each compound was studied for the following responses (i.e., dependent variables): pH and contact angle formed between a stationary drop of the formulation and the beef cut surfaces. The selected Ch concentration range was based on previous studies performed on meat samples [4,16,21,22,23].

Ch solutions were prepared by dissolving the biopolymer in 1.0% (*v*/*v*) lactic acid aqueous solution containing 2% GTE under agitation for 1 h at 80 °C. The GTE concentration of 2% was selected based on previous studies [24,25] and preliminary tests. Then, only one essential oil (i.e., TO, FO or OO) was added separately to different Ch formulations and homogenized using an Ultra-Turrax homogenizer (T18, IKA-Werke, Staufen, Germany) at 16,000 rpm for 5 min at 20 °C to produce a stable solution. More information regarding coating formulations is available in Table 1 and Table S1.

Based on the response surfaces and contour curves (for pH and contact angle values) described and discussed in Section 3.1.1, one Ch–GTE–FO formulation was selected for beef application, since coating solutions containing OO or TO were not able to efficiently cover the meat surface. In addition, the FO selection was economically advantageous because the TO and OO cost was approximately 100 times higher than FO according to information provided by Celeiro (Portugal). All experimental design analyses were performed by the Protimiza software (http://experimental-design.protimiza.com.br (accessed on 1 September 2022)).

### 2.4. Characterization of Coating Formulations

#### 2.4.1. pH and Contact Angle Measurements

The pH of the coating formulations was measured using a digital pH meter (Hanna Instruments, Inc., Nusfalau, Romania) equipped with a surface probe previously calibrated with buffer solutions pH 4.0 and 7.0. The probe was dipped directly into the coating solutions.

In order to obtain uniform spreading on the beef surface, the contact angle formed between coating formulations and beef cuts’ surfaces was determined for each coating. The contact angle at the beef surface was measured by the sessile drop method and observed with a contact angle meter (OCA 20, Dataphysics, Filderstadt, Germany) by computer-aided image processing using a digital camera. The coating solutions at different constituent concentrations were applied with a 500 µL automatic piston syringe (Hamilton, Switzerland) with a 0.75 mm diameter needle. To avoid changes on the beef surface, measurements were made in less than 15 s [26]. Six replicates of contact angle measurements were obtained at 22 ± 1.8 °C for each Sh, St and Kn sample.

#### 2.4.2. Rheological Parameters

##### Flow Curves

Rheological properties of coating solutions were obtained using an Instruments HR1 rheometer equipped with a Peltier plate (TA Instruments, New Castle, NSW, USA) with a stainless-steel cone-plate geometry (6.0 cm, 2° angle, truncation 67 μm). All the measurements were performed at 25 °C. Flow curves were obtained by an up–down–up step program using different shear stresses to provide shear rates ranging from 0 to 300 s^−1^. Newtonian (Equation (1)) and power-law (Equation (2)) models were fitted to the data to obtain rheological properties.

(1) $$\sigma =\eta \times \dot{\gamma }$$

(2) $$\sigma =k\times {\dot{\gamma \;}}^{n}$$

where *σ* is the shear stress (Pa), η is the viscosity (Pa.s), k is the consistency index (Pa.s*^n^*), $\dot{\gamma }$ is the shear rate (s^−1^) and *n* is the flow index.

##### Oscillatory Measurements

The viscoelastic properties of the coating formulations were evaluated using a frequency sweep between 0.1 and 10 Hz within the linear viscoelasticity domain. The contributions of the elastic and viscous characteristics were analyzed from storage (*G′*) and loss (*G″*) moduli, respectively. The loss tangent (tan δ) was evaluated to define the prevailing behavior as elastic or viscous. Values of tan δ < 1 indicate a predominantly elastic behavior, whereas tan δ > 1 denotes a predominantly viscous behavior that is directly related to the energy lost per cycle divided by the energy stored per cycle (Equation (3)).

(3) $$tan\delta =\frac{G\prime \prime }{G\prime }$$

where *δ* is the phase angle between the applied strain and the stress response.

### 2.5. Application of Optimized Coating Formulation on Beef Cut Samples

After the selection of the optimized coating formulation (2% Ch + 2% GTE + 0.1% FO), according to the pH, contact angle and rheological properties, the beef cuts (Sh, St and Kn) were dipped in this solution for 5 s and then the coated samples were air-dried for 30 s during the cutting meat process day. Then, the coated beef cuts (Ch–Sh, Ch–St and Ch–Kn) and non-coated samples (n/Ch–Sh, n/Ch–St and n/Ch–Kn) were vacuum-packed inside polyethylene bags using a GK852B Supervac machine (Austria) and stored in a cold storage room at 4 °C for 8 days.

### 2.6. Shelf-Life Analysis

The shelf-life analyses were performed at 0 (T0), 5 (T5) and 8 (T8) days after the application of the optimized Ch–GTE–FO coating formulation on beef cuts samples. The evaluation of microbiological, physicochemical and mechanical parameters was conducted as described below.

#### 2.6.1. Microbiological Analysis

Microbiological analyses of beef cut samples on tryptone glucose extract agar were carried out for total viable count (TVC) after incubation at 30 °C for 2 days according to EN ISO 4833-1:2013 and for *Enterobacteriaceae* counts on Violet Red Bile Dextrose agar after incubation at 37 °C for 2 days according to ISO 21528-2:2017. The effect of the coating on the microbial count of meat samples stored under refrigerated conditions was evaluated during storage at days 0, 5 and 8. All counts were expressed as log colony-forming units (log CFU.g^−1^).

#### 2.6.2. Mechanical Properties

A texture profile analysis (TPA) was performed on raw beef cuts according to previous studies [22,27,28] to obtain six textural parameters: hardness, firmness, resilience, springiness, gumminess, adhesiveness and cohesiveness. A TA.HDPlus Texture Analyser (Stable Micro Systems Ltd., Godalming, UK) equipped with a load cell of 250 kg was used to obtain the force–time deformation curves. A 2-cycle compression test was performed at up to 80% (strain) compression of the initial height with a 20 mm diameter plate and 20% (strain) compression for the second cycle at a test speed of 5 mm/s. A time of 5 s was allowed to elapse between the first and second compression cycles. The parameters retained with this test were the peak positive force of the first cycle, the area to positive peak of the first and second cycles and the distance of the first and second cycles (from the beginning to the maximum peak—obtained by manually marking in the texturometer exponent software the points from the beginning to the top of a peak), which were used to calculate all the texture parameters. Each coated and non-coated beef cut sample (Sh, St, and Kn) was tested in triplicate at 21 °C. For data analysis, the Texture Exponent version 6.1.1.0 software by Stable Microsystems (Surrey, UK) was used.

#### 2.6.3. Colorimetric Parameters

Color evaluation of beef samples were assessed by the CIELAB color system using the L*a*b* coordinates, where L* is the luminosity, varying from black (0%) to white (100%); a* is the intensity of the red color, varying from green (−a) to red (+a); and b* the intensity of the yellow color, varying from blue (−b) to yellow (+b). These measures were performed with a colorimeter (Minolta CR-300, Chromometer, Osaka, Japan) provided with a diffuse illumination, 0° standard viewing geometry, D65 illuminant, 8 mm diameter aperture and calibrated against a white tile (provided by the manufacturer) [29]. The changes in beef color were determined after the packaging film was removed and over a 30 min bloom time period [30]. The average value of L*, a*, and b* (at least three measurements obtained in each replicate) were used for statistical analysis. The total color differences (∆E*) in coated and non-coated beef cut samples were estimated by Equation (4):

(4) $${\Delta E}^{*}=\sqrt{{\left({\Delta L}^{*}\right)}^{2}+{\left({\Delta a}^{*}\right)}^{2}+{\left({\Delta b}^{*}\right)}^{2}}$$

#### 2.6.4. pH Value

The pH values of the beef cut samples were measured using a digital pH meter (Hanna Instruments, Inc., Romania, Balkans) equipped with a surface probe previously calibrated with buffer solutions pH 4.0 and 7.0. The analysis was performed by the direct contact of the probe with three different points of the surface of each beef cut.

#### 2.6.5. Lipid Oxidation (TBARS)

The lipid oxidation assessment was conducted by the thiobarbituric acid-reactive substances (TBARS) test according to methodologies proposed by Raharjo et al. (1992) [31] and Wang et al. (2002) [32], with some modifications. Initially, 0.5 mL of BHT (0.5%) was added to a tube containing 5 g of sample (which corresponds to small slices of the whole beef cut). Afterward, 18 mL of TCA (5%) was added to each tube sample and vortexed for 1 min. The mixture was filtered, and 2 mL of the filtrate was mixed with 2 mL of reagent TBA and placed in a water bath at 80 °C for 40 min. Then, the absorbance readings were performed at 531 nm in 96-well plates using the Synergy™ HT Multi-detection Microplate Reader (Bio-Tek Instruments, Inc., Winooski, VT, USA). The analysis was performed in triplicate and the results were expressed in mg of malonaldehyde (MDA). kg^−1^ of sample.

### 2.7. Statistical Analysis

All data were expressed as mean ± standard deviation (SD). OriginPro2018^®^ Statistic Software (Origin Lab Corporation, Northampton, MA, USA) was used for data analysis. To assess the effects of coating treatments and storage time on the microbial quality and physicochemical characteristics of the beef cut samples, a one-way analysis of variance (ANOVA) was performed. Statistical analysis was conducted comparing the equivalent beef cut exposed to different treatments (i.e., coated or non-coated sample) and to different storage times (i.e., T0, T5 and T8). For each storage time, at least three beef samples from each treatment group were evaluated. The study was replicated for validation under the same conditions. Means were compared using Tukey’s Test. Differences among the mean values were considered significant when *p* < 0.05.

## 3. Results and Discussion

### 3.1. Selection of Coating Formulations for Beef Application

#### 3.1.1. pH and Contact Angle

In order to maintain the initial characteristics of the studied beef samples, one of the considered points when choosing the coating to be applied was the pH of the solution (i.e., pH = 5.8 ± 0.2). As can be seen in Table 2, only the axial point corresponding to the lowest concentration of Ch had a significantly different pH compared with the pH of the beef samples. The final formulation was selected after performing contact angle analysis, in order to ensure that there was a balance between adhesion and absorption by the beef surface. In general, Ch formulations with 2% GTE presented contact angles with low magnitude, resulting in a high absorption of the coating formulation in the beef (Supplementary Material; Table S1).

**Figure 1:**
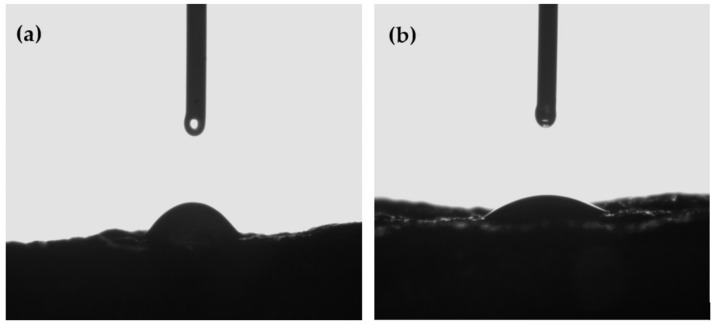
Image of contact angle measurements of the optimized formulation 2% Ch + 2% GTE + 0.1% FO (**a**) and 2% Ch + 2% GTE + FO 0.03% (**b**) applied on the beef surface.

Results showed that a concentration of Ch ≥ 2% plus a concentration of FO ≥ 0.03% (i.e., formulations 2% Ch + 0.03% Ch, 2% Ch + 0.1% FO, and 2.31% Ch + 0.06%) presented higher contact angle values. The 2% Ch + 0.03% Ch solution was rejected because of the significantly lower contact angle value obtained compared with 2% Ch + 0.1% FO and 2.31% Ch + 0.06% formulations, as can be seen in Table 2 and Figure 1. On the other hand, the addition of 0.1% and 0.06% of FO to 2% and 2.31% of Ch aqueous solution, respectively, balanced the cohesive and adhesion forces, resulting in a contact angle of 22.3° ± 0.4 and 23.7° ± 0.5, respectively. This result demonstrated the coating capacity to spread evenly on the beef surface (Table 2). Thus, an increase in FO concentration improved compatibility between the solution and the beef surface, as can be seen in Figure 1. Moreover, a higher FO concentration (i.e., 0.1% FO instead of 0.06%) possibly increases coating formulation functionality (i.e., antioxidant activity), which, according to previous studies, could be an advantage for beef preservation [33]. Based on these results, the 2% Ch + 2% GTE + 0.1% FO formulation was selected for further analysis.

#### 3.1.2. Rheological Parameters

**Figure 2:**
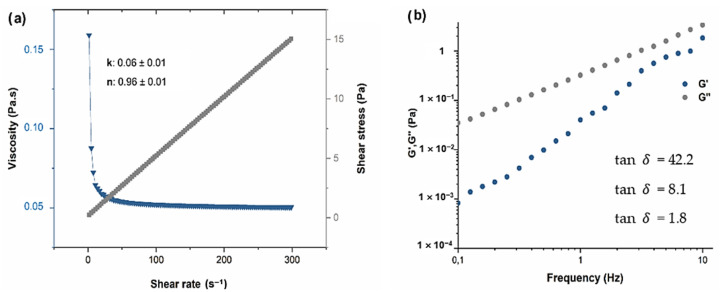
(**a**) Flow curve (gray symbols) and respective viscosity (blue symbols) of the chitosan optimized formulation (2% Ch + 2% GTE + 0.1% FO) in a shear rate range of 0 to 300 s^−1^ under isothermal (25 °C) conditions and its flow (*n*) and consistency (*k*) indexes; (**b**) Elastic modulus (blue symbols) and viscous modulus (grey symbols) as a function of frequency (0.1 to 10 Hz) under isothermal (25 °C) conditions and tan δ at different frequencies for the formulation 2% Ch + 2% GTE + 0.1% FO.

Figure 2a shows that the flow curve presented a non-Newtonian (R^2^ = 0.99) shear-thinning behavior, as the *n* value was lower than 1 (*n* = 0.96). This result implies a decrease in the apparent viscosity at increased shear rates, although the Newtonian fluid model was also well fitted (R^2^ = 0.96) as seen from the very low pseudoplasticity and low consistency index (0.06) presented. The apparent viscosity is shown in Figure 2a, where it is possible to observe an abrupt decrease in viscosity up to a shear rate of 20 s^−1^, followed by a maintenance of viscosity around 0.05 Pa.s.

In addition to these low viscosities values, a predominantly viscous character (liquid-like) was observed through the oscillatory tests, since the loss modulus (*G″*) was always higher than the storage modulus (*G′*) (tan*δ* > 1) in the interval from 0.1 to 10 Hz (Figure 2b). These facts indicate that, besides being applied by a dipping process, this formulation can be easily applied in beef by spraying processes since the force/tension required to make the coating flow through a nozzle (with micro dimensions) will be low.

### 3.2. Shelf-Life Evaluation

#### 3.2.1. Microbiological Analysis

**Figure 3:**
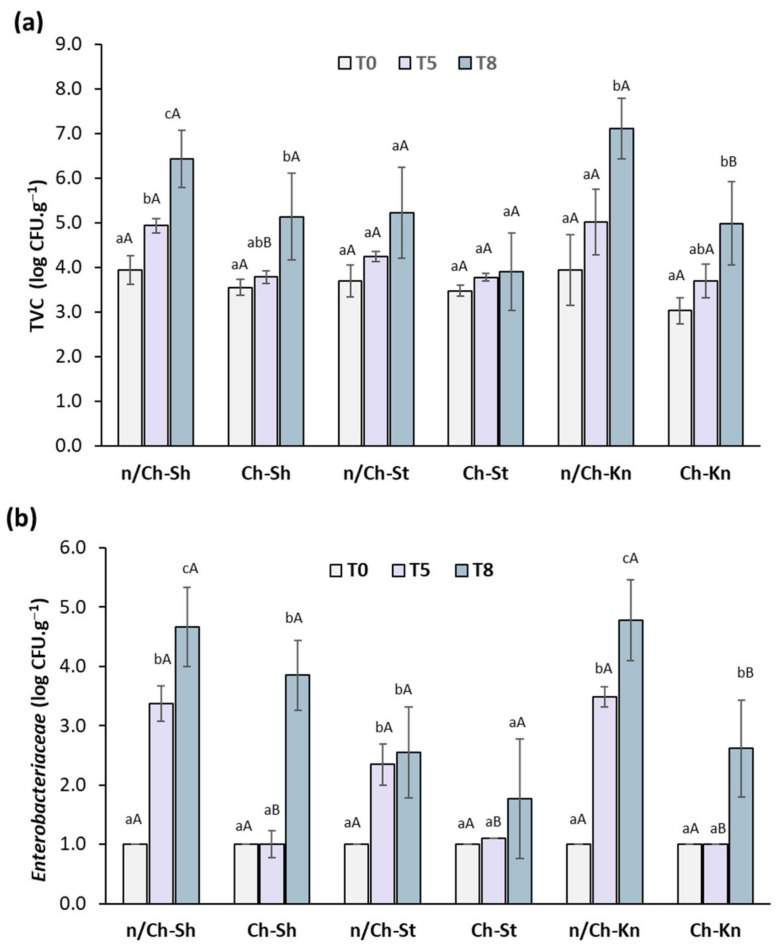
TVC (**a**) and *Enterobacteriaceae* count (**b**) of vacuum-packed beef cut samples over the storage period of 0 (T0), 5 (T5) and 8 (T8) days at 4 °C. n/Ch–Sh = non-coated shoulder; Ch–Sh = coated shoulder; n/Ch–St = non-coated striploin; Ch–St = coated striploin; n/Ch–Kn = non-coated knuckle; Ch–Kn = coated knuckle. Columns represent the mean (*n* = 3) and the bars represent the standard deviation. ^a–c^ Different letters indicate significant differences (*p* < 0.05) for the same beef cut sample over the storage period (T0, T5 and T8). ^A,B^ Significant differences (*p* < 0.05) between the different beef cuts on the same storage day.

Effects of the Ch–GTE–FO coating on the TVC in beef samples during refrigerated storage are shown in Figure 3a. As can be seen, TVC increased in all treatment groups (coated and non-coated samples) over storage time (*p* < 0.05) but at different rates. This result is more apparent on the TVC values of the n/Ch–Sh samples, which increased at a faster rate than those of Ch–Sh samples from T0 to T5 (*p* < 0.05), indicating that the coating treatment exhibited a bacteriostatic effect. It has been reported that the maximum acceptable microbial limit for good quality beef is 6 log CFU.g^−1^ [34]. This limit was overcome after 8 days of storage for n/Ch–Sh and n/Ch–Kn samples. However, the TVC values of the other samples were acceptable at T8 (Figure 3a). Thus, these results demonstrated that the combined use of Ch, GTE and FO as a coating on beef samples delays the growth of TVC in beef samples stored up to 8 days under cold storage. It is noteworthy that the concentrations of the compounds used in the study were effective in this case. However, the microbial growth inhibition of the developed coating disappeared with increasing storage time. As previously mentioned in other works, Ch has the capacity to reduce bacterial load on meat products due to its positive charge (free amino groups), which interferes with the microbial cell membranes (negative charge), resulting in cell membrane leakage [35]. The lactic acid used to dissolve chitosan could also influence its effectiveness in controlling the microorganisms on the beef samples during storage. It was previously reported that organic acids such as lactic acid present antimicrobial activity [36]. In addition, GTE was reported to present antimicrobial activity due to the action of catechins which bind to the microorganism’s lipid bilayer membrane, causing its damage [37]. Our results are in accordance with another study, where minced beef samples treated with a chitosan–kombucha tea coating showed a delay of microbial growth compared to non-coated samples during storage [38].

*Enterobacteriaceae* enumeration is usually a quality parameter used to assess hygiene and sanitation in the meat processing industry [39]. The T0 analyses of all beef samples showed that *Enterobacteriaceae* counts were below 1 log CFU/g (Figure 3b). However, the *Enterobacteriaceae* counts in the coated beef samples were significantly lower (*p* < 0.05) than non-coated beef samples at day 5 (T5) of storage. Thus, the coating demonstrated a protective effect against the contamination process by *Enterobacteriaceae*. At the T8 storage timepoint, an increase in *Enterobacteriaceae* count was observed for coated and non-coated beef samples. These results are in agreement with the Langroodi et al. (2021) [40] study which showed that a chitosan–grape seed extract coating on turkey breast meat had an antibacterial effect against *Enterobacteriaceae*. Other work also reported that *Enterobacteriaceae* growth was inhibited by chitosan–cinnamon essential oil in beef patties during storage and under refrigeration conditions [41].

#### 3.2.2. Textural Properties

Texture parameters of coated and non-coated beef cut samples during storage at 4 °C under vacuum conditions are shown in Table 3.

The hardness parameter is highly influenced by the amount of water present in the beef sample. It was expected that coated samples presented a lower hardness value compared to non-coated samples because the Ch coating should be acting as a barrier to water loss, as already mentioned in other works where Ch was applied to perishable food such as meat, fish and fruits [26,42,43,44]. However, no significant differences between coated and non-coated beef cut samples (*p* > 0.05) were observed for the hardness parameter, showing that sample tenacity did not change during storage time. It is important to mention that the beef samples were also vacuum-packed which possibly contributed as a barrier effect against water content loss in all samples.

Regarding the adhesivity parameter, significant differences (*p* < 0.05) were observed between T0 and T8 for n/Ch–Kn and also for n/Ch–St samples. An increase in n/Ch–Kn adhesiveness values was observed, resulting in a sticky beef sample (i.e., high adhesive force and low cohesive force), since the cohesion parameter did not change during storage time (T0 to T8). This fact can be associated with the significant increase in the microbial load for those cut sample types (Figure 3), causing a decrease in the integrity of muscle tissue caused by microorganisms and, thus, being a determining factor of beef quality [22]. On the other hand, adhesiveness of the n/Ch–St sample decreased (i.e., less sticky surface), which can be correlated to changes of the myofibrillar component during cold storage and to the minimal amount of fat present in this type of cut over storage time [45].

The springiness parameter demonstrates how long beef samples will take to reach their original shape and mechanical properties after compression. The springiness value of Ch-coated beef samples did not change significantly (*p* > 0.05) during storage. Springiness values are also related to the elastic properties of the raw beef cut samples. The decrease in the springiness value indicates sample elasticity loss [46]. Since the springiness value of the n/Ch–St sample was significantly lower (*p* < 0.05) at T5 and T8 than at T0, this demonstrated that this sample is more sensitive to deformation speed. As for the n/Ch–Sh sample, its springiness value was higher at T8, thus providing higher resistance to deformation. This result can be directly correlated with the chewiness parameter, since significant differences (*p* < 0.05) were detected in n/Ch–Sh and n/Ch–St samples after 8 days. As result, the required energy to deform the n/Ch–Sh sample increased because of its elastic resistance in contrast to the n/Ch–St sample [47]. Although chewiness parameter determination was carried out on raw beef, this result may be associated with higher difficulty in chewing the cooked beef [42,45,48].

The resilience of the studied beef cuts is comparable in all cases except for n/Ch–St at T8, which shows significant lower values (*p* < 0.05). This result indicates that the n/Ch–St sample presented less ability to recover its height, deforming permanently after the first loading cycle [49]. Since this parameter represents a measurement of how the sample recovers from deformation, it provides confirmation of the significantly lower elasticity of the n/Ch–St at T8, which is also reflected in its low springiness value [50].

Gumminess is calculated using hardness and cohesiveness, which represents the resistance to a compressive force and the respective ability to retain its form between the 1st and 2nd cycle. As for the gumminess, no significant differences (*p* > 0.05) were observed for the different beef samples at different storage times, indicating that the same energy is required to disintegrate the Sh, Kn and St cuts [46]. This fact is in accordance with the hardness and cohesion parameters, since no significant differences were observed (*p* > 0.05).

To conclude, it is important to mention that all the textural parameters (i.e., hardness, adhesiveness, cohesion, resilience, springiness, gumminess and chewiness) were maintained during the 8 days after Ch–FO–GTE coating application plus vacuum-packaging; this is possibly a result of the Ch, FO and GTE action against enzymatic and microbiological processes, which avoids significant textural changes to the beef samples [28,50].

#### 3.2.3. Colorimetric Parameters

One of the key features which influence the purchase of fresh meat is its color, as the consumer perceives meat freshness based on its red color [35].

**Figure 4:**
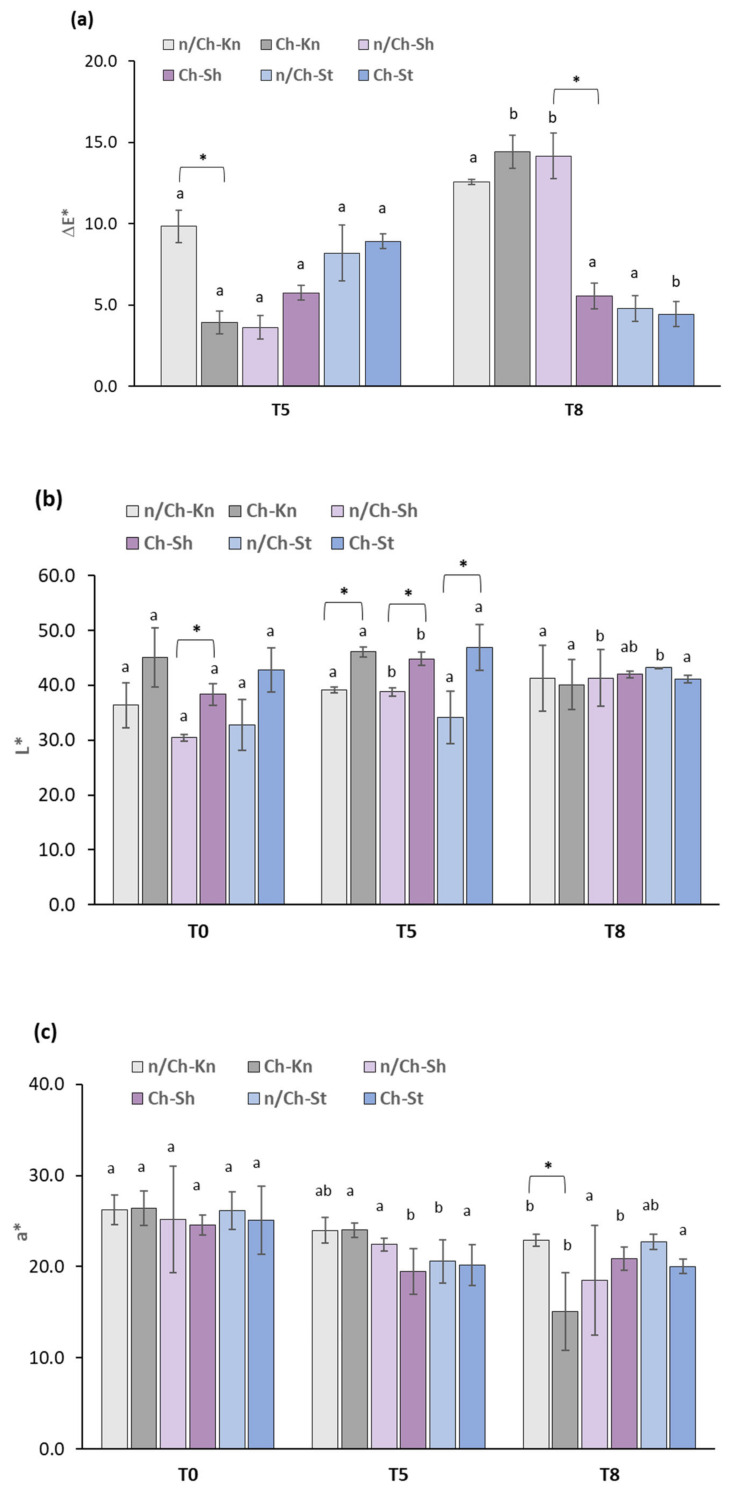

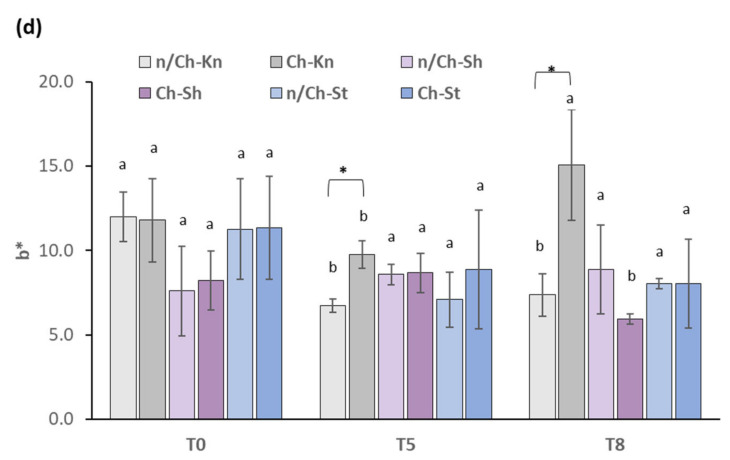
Color properties—(**a**) ∆E*, (**b**) L*, (**c**) a* and (**d**) b*—of vacuum-packed non-coated and coated beef cut samples over the storage period of 0 (T0), 5 (T5) and 8 (T8) days at 4 °C. n/Ch–Sh = non-coated shoulder; Ch–Sh = coated shoulder; n/Ch–St = non-coated striploin; Ch–St = coated striploin; n/Ch–Kn = non-coated knuckle; Ch–Kn = coated knuckle. Columns represent the mean (*n* = 3), and the bars represent the SD. ^a,b^ Different letters indicate significant differences (*p* < 0.05) for the same beef cut sample and with the same treatment over the storage period (T0, T5 and T8). * Significant differences (*p* < 0.05) between the same beef cut with different treatments (non-coated and coated samples) on the same storage day.

Thus, the effects of coating on the color properties (∆E*, L*, a* and b*) of beef samples under vacuum-packaging conditions during refrigerated storage were assessed (Figure 4). According to ∆E* results, changes in the visual color of the samples could be clearly identified by an observer because ∆E* > 3.5 [51]. Some color change differences were detected between coated and non-coated beef samples over time (Figure 4a). For instance, n/Ch–Kn showed higher ∆E* color changes than Ch–Kn on day 5 of storage (*p* < 0.05) which showed that Ch–GTE–FO coating postponed beef discoloration over 5 days, possibly due to the capacity of Ch, GTE and FO to scavenge the radicals generated in meat [52]. However, Ch–Kn samples displayed increased ∆E* values on day 8, and the gap between n/Ch–Kn and Ch–Kn was gradually reduced, which was not accompanied by any significant differences (*p* > 0.05). On the other hand, the Ch–Sh sample showed lower ∆E* values on day 8 than the n/Ch–Sh sample (*p* < 0.05), possible due to the coating’s ability to minimize lipid oxidation in the Sh sample which is in accordance with the TBARS results obtained in this study (Section 3.2.5).

Both non-coated and coated beef samples maintained stable lightness (L*) values over the storage period (Figure 4b). However, all coated samples (Ch–Kn, Ch–Sh and Ch–St) displayed a significant increase in L* values (lightness) on day 5 compared to uncoated samples (Figure 4b). A possible cause for these results may be due to the glossy nature of the Ch–GTE–FO coating and its interactions with the moisture layer on the beef surface.

Overall, coating treatments did not significantly influence the beef redness (a*) and yellowness (b*) compared to non-coated samples over 8 days of storage (Figure 4c,d). This could be attributed to no significant changes being observed in lipid oxidation among the beef samples (Section 3.2.5) and also to the outcome of vacuum packaging and refrigerated storage, which maintain stable a* and b* values. Likewise, Gedarawatte et al. (2022) [53] reported minimal change of a* and b* values of vacuum-packaged beef samples coated with nisin and bacterial cellulose nanocrystals during 21 days of storage. However, the Ch–Kn sample showed a significant reduction in the a* value and a rise in the b* value (*p* < 0.05) compared with n/Ch–Kn after 8 days of storage (Figure 4c,d).

In this case, the decrease in Ch–Kn redness and the increase in its yellowness may be related to the myoglobin chemistry and specific fiber structure of the Kn. Possibly, coating decreased oxygen penetration and consequently, myoglobin oxygenation (i.e., metmyoglobin formation, a brown oxidized form of myoglobin) which decreased the red color in the Ch–Kn sample [54,55]. Moreover, other factors could also be responsible for color changes observed in the Ch–Kn sample, namely, Kn microstructure (e.g., muscle fiber types and myofibrillar structure), as previously reported [56]. Further studies will be necessary to determine the influence of these factors on beef color.

#### 3.2.4. pH Value

**Figure 5:**
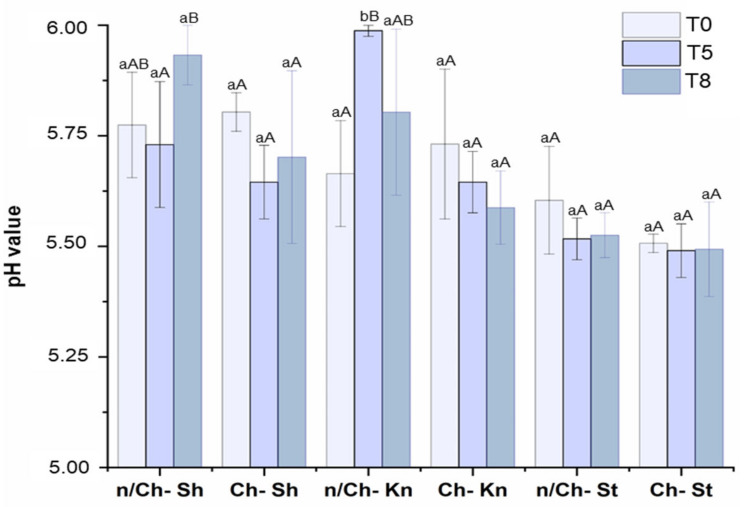
pH values of vacuum-packed non-coated and coated beef cuts samples over the storage period of 0 (T0), 5 (T5) and 8 (T8) days at 4 °C. n/Ch–Sh = non-coated shoulder; Ch–Sh = coated shoulder; n/Ch–St = non-coated striploin; Ch–St = coated striploin; n/Ch–Kn = non-coated knuckle; Ch–Kn = coated knuckle. Columns represent the mean (*n* = 3), and the bars represent the standard deviation. ^a,b^ Different lowercase letters show significant differences (*p* < 0.05) between the same beef cut with different treatments on the same storage day. ^A,B^ Different uppercase letters indicate significant differences (*p* < 0.05) for the same beef cut sample over the storage period (T0, T5 and T8).

The pH value of meat products is an important parameter to be monitored during their storage as it is related to sensory properties such as juiciness, freshness, flavor, color, and tenderness, among others [57,58]. Moreover, changes in pH values can help us understand the damage that occurs in food matrices due to microbial growth and the decomposition of proteins [57,59]. The pH values of vacuum-packed non-coated and coated beef cut samples over the storage period (T0, T5 and T8) at 4 °C are shown in Figure 5.

During the storage period, all coated beef cut samples, and n/Ch–St showed similar pH values (*p* < 0.05), while n/Ch–Sh and n/Ch–Kn showed pH value oscillation (*p* < 0.05). There were no significant differences (*p* < 0.05) in the pH values among the coated and non-coated samples at T0. However, n/Ch–Sh and n/Ch–Kn showed an increase (*p* < 0.05) of pH values at T5 and T8, respectively.

The maintenance of pH in coated beef samples during storage can be attributed to the protective Ch activity against microbial growth [60], which is reinforced by the microbial count presented in Figure 3. Similar results were obtained by Hoa et al. (2022) [35], who reported that the pH increase in the beef samples was retarded by Ch coating. Several studies have reported an increase in pH value of non-coated meat compared with coated meat at the end of storage. The authors attributed the increase in pH to the accumulation of bacterial metabolites resulting from microbial proliferation [57,61] and also to the natural acidification of coating components [62].

#### 3.2.5. Lipid Oxidation (TBARS)

**Figure 6:**
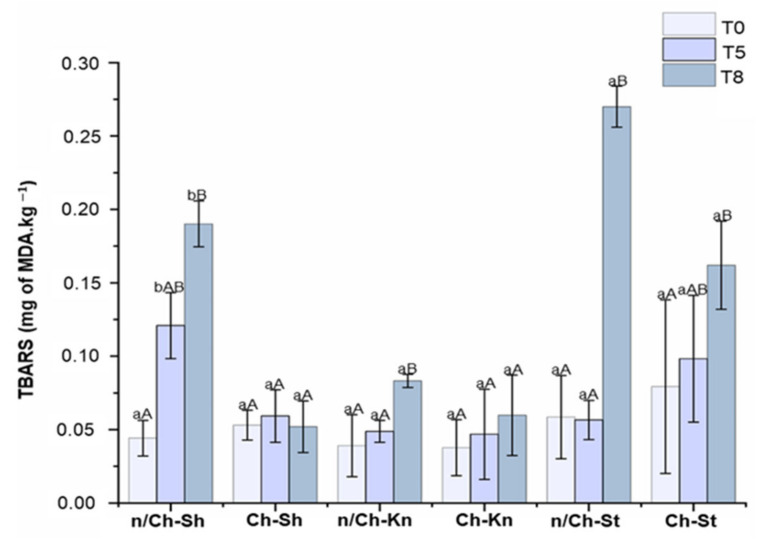
Thiobarbituric acid-reactive substances (TBARS) (mg of malonaldehyde (MDA)/kg of meat) of vacuum-packed beef cut samples (non-coated and coated) over the storage period of 0 (T0), 5 (T5) and 8 (T8) days at 4 °C. n/Ch–Sh = non-coated shoulder; Ch–Sh = coated shoulder; n/Ch–St = non-coated striploin; Ch–St = coated striploin; n/Ch–Kn = non-coated knuckle; Ch–Kn = coated knuckle. Columns represent the mean (*n* = 3), and the bars represent the standard deviation. ^a,b^ Different lower case letters show significant differences (*p* < 0.05) between the same beef cut with different treatments on the same storage day. ^A,B^ Different uppercase letters indicate significant differences (*p* < 0.05) for the same beef cut sample over the storage period (T0, T5 and T8).

Lipid oxidation is one of the major factors that cause meat quality loss, off-flavors and discoloration [34,63]. Accelerated by factors such as oxygen, iron, light and temperature, the lipid oxidation in meat is commonly evaluated by the TBARS assay, which measures the MDA content (a main secondary product formed in the process) [34]. The TBARS values of vacuum-packed non-coated and coated beef cuts samples expressed as MDA content (mg.kg^−1^) over the storage period at 4 °C are shown in Figure 6.

According to Possamai et al. (2018), TBARS values over 2 mg of MDA.kg^−1^ represent a perceptible and unacceptable off-odor in meat [64]. Thus, all analyzed samples possibly did not present a perceptible off-odor because the TBARS values obtained were below 2 mg of MDA.kg^−1^ at the end of storage. However, differences were found between non-coated and coated samples. Coated treatment promoted a significant (*p* < 0.05) inhibition of lipid oxidation on Ch–Sh after five days of storage. The effect of coating was more pronounced when lipid oxidation was compared during the full storage time (T0 to T8). All non-coated samples showed a significant (*p* < 0.05) increase in lipid oxidation at the end of storage (T8). Regarding coated samples, only Ch–St showed a significant (*p* < 0.05) increase in lipid oxidation at T8, while Ch–Sh and Ch–Kn maintained similar lipid oxidation values (*p* < 0.05) over the storage time (Figure 6). The low TBARS value of coated beef cut samples found in our study can be explained by the effect of coated components on lipid oxidation. Ch, when applied as an edible coating, exhibits oxygen barrier capability and antioxidant properties, playing an important contribution to retarding the lipid oxidation of beef products [65]. Additionally, the incorporation of the GTE and FO enhanced the antioxidant properties of the coating [16,66], since they are a good source of phenolic compounds that act as free radical scavengers to terminate the radical chain reactions that occur during lipid oxidation [67]. Thus, the results found in our study suggest that the Ch coating reduced the access and diffusion of oxygen to the beef surface, and that the phenolic compounds present in GTE and FO chelated free radicals, delaying the lipid oxidation process.

Similar to our study, lower TBARS values during storage were also reported in Ch-coated pork [68,69], fish [70,71] and beef [35,60,72] compared to non-coated meat. In addition, coatings incorporating GTE presented lower TBARS than non-coated samples [73,74]. For instance, Wrona et al. (2021) [33] evaluated the antioxidant capacity of films containing different essential and vegetable oils. The authors reported that the storage of foods prone to oxidation could be improved by using films with FO, which have extended fresh meat shelf life by 22%.

## 4. Conclusions

The results obtained in this study indicated that FO presented a higher potential than OO and TO to be used in Ch–GTE coating formulations, promoting a balanced contact angle value when applied on the beef cuts’ surface and, consequently, covering homogeneously the beef surface. The optimised coating formulation (2% Ch + 2% GTE + 0.1% FO) showed the ability to be applied to beef by dipping or spraying, as demonstrated by rheological properties (i.e., low viscosity, low consistency index, low pseudoplasticity and the absence of yield stress).

The combined use of Ch–GTE–FO has been demonstrated to delay microbial growth, lipid oxidation and color changes in beef cut samples (St, Sh and Kn), as well as maintain textural parameters during 8 days of storage at 4 °C, thus increasing beef shelf-life. Overall, the results obtained in this study demonstrate the enormous potential of Ch–GTE–FO as an active packaging on preserving fresh beef cuts’ quality during refrigerated storage along with vacuum packaging. However, further research is still required to assess the influence of Ch–GTE–FO coating application on the sensorial attributes and, consequently, sensorial acceptance of coated-beef cuts.

## Figures and Tables

**Table 1:** Composition of chitosan (Ch) and essential oil (TO, FO or OO) coating formulations containing 2% GTE according to the CCRD matrix.

Ch (% *w*/*v*)	Essential Oil (% *w*/*v*)
0.5 (−1) ^a^	0.03 (−1)
2 (1)	0.03 (−1)
0.5 (−1)	0.1 (1)
2 (1)	0.1 (1)
0.19 (−1.41)	0.06 (0)
2.31 (1.41)	0.06 (0)
1.25 (0)	0.01 (−1.41)
1.25 (0)	0.12 (1.41)
1.25 (0)	0.06 (0)
1.25 (0)	0.06 (0)
1.25 (0)	0.06 (0)

^a^ Coded (in parenthesis) and uncoded values represent components’ concentration.

**Table 2:** pH and contact angle of the Ch–GTE (2%) coating solution formulations containing FO according to the CCRD matrix.

Biopolymer	Concentration (%)	Essential Oil	Concentration (%)	pH	Contact Angle (°)
Chitosan	0.5	Flaxseed	0.03	5.5 ± 0.1	16.9 ± 0.1
	2		0.03	5.9 ± 0.1	19.6 ± 0.2
	0.5		0.1	5.5 ± 0.1	17.1 ± 0.2
	2		0.1	5.8 ± 0.1	22.3 ± 0.4
	0.19		0.06	5.3 ± 0.0	14.9 ± 0.1
	2.31		0.06	5.8 ± 0.1	23.7 ± 0.5
	1.25		0.01	5.6 ± 0.1	17.9 ± 0.2
	1.25		0.12	5.7 ± 0.1	17.1 ± 0.3
	1.25		0.06	5.8 ± 0.0	19.1 ± 0.2
	1.25		0.06	5.7 ± 0.1	18.9 ± 0.3
	1.25		0.06	5.8 ± 0.0	19.0 ± 0.1

**Table 3:** Texture parameters (hardness, adhesiveness, resilience, cohesion, springiness, gumminess and chewiness) obtained for the different coated and non-coated beef cuts (Sh, Kn and St) on days 0, 5 and 8 days.

Time (days)	Sample	Hardness (kg)	Adhesiveness (kg.s)	Resilience (%)	Cohesion	Springiness (%)	Gumminess	Chewiness
0	n/Ch–Sh	10.93 ± 1.65 ^a^	−94.54 ± 24.34 ^a^	13.89 ± 1.78 ^a^	0.21 ± 0.04 ^a^	34.60 ± 4.94 ^a^	2.31 ± 0.53 ^a^	0.81 ± 0.20 ^a^
	n/Ch–Kn	12.10 ± 1.18 ^a^	−79.97 ± 15.93 ^a^	12.82 ± 2.30 ^a^	0.21 ± 0.04 ^a^	26.97 ± 4.69 ^a^	2.56 ± 0.47 ^a^	0.70 ± 0.15 ^a^
	n/Ch–St	12.05 ± 1.01 ^a^	−65.25 ± 17.01 ^a^	9.58 ± 1.00 ^a^	0.18 ± 0.04 ^a^	37.19 ± 5.09 ^a^	2.20 ± 0.35 ^a^	0.82 ± 0.16 ^a^
5	n/Ch–Sh	12.31 ± 1.69 ^a^	−108.50 ± 29.90 ^a^	13.07 ± 3.09 ^a^	0.22 ± 0.05 ^a^	37.64 ± 4.43 ^a^	2.60 ± 0.50 ^a^	0.91 ± 0.21 ^a^
	n/Ch–Kn	13.24 ± 1.89 ^a^	−88.50 ± 9.84 ^a^	10.25 ± 2.84 ^a^	0.19 ± 0.03 ^a^	35.94 ± 4.80 ^a^	2.61 ± 0.29 ^a^	0.93 ± 0.15 ^a^
	n/Ch–St	11.24 ± 2.32 ^a^	−48.27 ± 3.79 ^a^	9.63 ± 3.40 ^a^	0.21 ± 0.06 ^a^	26.63 ± 4.51^b^	2.26 ± 0.57 ^a^	0.61 ± 0.24 ^a,b^
	Ch–Sh	12.64 ± 2.06 ^a^	−65.05 ± 20.41 ^a^	14.88 ± 2.63 ^a^	0.27 ± 0.06 ^a^	34.06 ± 5.09 ^a^	2.75 ± 0.53 ^a^	1.09 ± 0.12 ^a^
	Ch–Kn	12.06 ± 2.59 ^a^	−61.75 ± 8.95 ^a^	11.39 ± 3.51 ^a^	0.20 ± 0.05 ^a^	27.14 ± 3.27 ^a^	2.33 ± 0.30 ^a^	0.62 ± 0.14 ^a^
	Ch–St	10.68 ± 1.94 ^a^	−56.46 ± 5.47 ^a^	9.76 ± 1.38 ^a^	0.20 ± 0.04 ^a^	30.88 ± 2.71 ^a^	2.12 ± 0.33 ^a^	0.63 ± 0.08 ^a^
8	n/Ch–Sh	9.92 ± 1.10 ^a^	−122.90 ± 23.97 ^a^	15.72 ± 2.85 ^a^	0.24 ± 0.07 ^a^	46.26 ± 5.74 ^b^	2.42 ± 0.61 ^a^	1.35 ± 0.19 ^b^
	n/Ch–Kn	11.27 ± 1.41 ^a^	−122.95 ± 19.73 ^b^	14.47 ± 2.65 ^a^	0.21 ± 0.03 ^a^	34.34 ± 4.57 ^a^	2.40 ± 0.40 ^a^	0.92 ± 0.09 ^a^
	n/Ch–St	14.28 ± 1.25 ^a^	−22.85 ± 2.74 ^b^	7.04 ± 0.52 ^b^	0.15 ± 0.02 ^a^	29.32 ± 0.96 ^b^	2.09 ± 0.11 ^a^	0.62 ± 0.05 ^b^
	Ch–Sh	11.65 ± 1.49 ^a^	−67.03 ± 9.70 ^a^	13.95 ± 2.72 ^a^	0.24 ± 0.05 ^a^	38.65 ± 4.11 ^a^	2.80 ± 0.59 ^a^	1.12 ± 0.37 ^a^
	Ch–Kn	10.40 ± 0.94 ^a^	−72.94 ± 17.54 ^a^	12.52 ± 2.97 ^a^	0.21 ± 0.05 ^a^	25.53 ± 3.87 ^a^	2.03 ± 0.53 ^a^	0.61 ± 0.14 ^a^
	Ch–St	11.27 ± 1.90 ^a^	−49.92 ± 14.32 ^a^	10.79 ± 3.41 ^a^	0.22 ± 0.05 ^a^	33.60 ± 4.90 ^a^	2.33 ± 0.69 ^a^	0.77 ± 0.06 ^a^

^a,b^ Different letters show significant differences (*p* < 0.05) for the same coated or non-coated beef cut samples over the storage period (0, 5 and 8 days).

## Data Availability

Data is contained within the article or Supplementary Materials.

## Supplementary Materials

The following supporting information can be downloaded at: https://www.mdpi.com/article/10.3390/foods12071447/s1, Table S1: pH and contact angle of the Ch–GTE coating solution formulations containing FO, TO, and OO, according to the CCRD matrix.
