# Supplementary material for: Chitosan Coating Functionalized with Flaxseed Oil and Green Tea Extract as a Bio-Based Solution for Beef Preservation
Source: Foods. 2023 Mar 29;12(7):1447. doi: 10.3390/foods12071447 (PMC10093991; doi:10.3390/foods12071447)
Supplement: Supplementary file 1 [file foods-12-01447-s001.zip › foods-2259811-supplementary.pdf]

Table S1. pH and contact angle of the Ch–GTE coating solution formulations containing FO, TO, and OO, according to the CCRD matrix.

Biopolymer	Concentration (%)	Essential Oil	Concentration (%)	pH	Contact angle (°)
	0.5		0.03	5.5 ± 0.1	16.9 ± 0.1
	2		0.03	5.9 ± 0.1	19.6 ± 0.2
	0.5		0.1	5.5 ± 0.1	17.1 ± 0.2
	2		0.1	5.8 ± 0.1	22.3 ± 0.4
	0.19		0.06	5.3 ± 0.0	14.9 ± 0.1
Chitosan	2.31	Flaxseed	0.06	5.8 ± 0.1	23.7 ± 0.5
	1.25		0.01	5.6 ± 0.1	17.9 ± 0.2
	1.25		0.12	5.7 ± 0.1	17.1 ± 0.3
	1.25		0.06	5.8 ± 0.0	19.1 ± 0.2
	1.25		0.06	5.7 ± 0.1	18.9 ± 0.3
	1.25		0.06	5.8 ± 0.0	19.0 ± 0.1
	0.5		0.03	5.5 ± 0.1	15.5 ± 0.2
	2		0.03	5.8 ± 0.1	17.3 ± 0.2
	0.5		0.1	5.4 ± 0.1	13.4 ± 0.3
	2		0.1	5.7 ± 0.1	14.9 ± 0.4
	0.19		0.06	5.4 ± 0.0	13.3 ± 0.5
Chitosan	2.31	Thyme	0.06	5.7 ± 0.1	18.2 ± 0.3
	1.25		0.01	5.7 ± 0.1	17.5 ± 0.4
	1.25		0.12	5.6 ± 0.1	14.9 ± 0.3
	1.25		0.06	5.6 ± 0.0	18.3 ± 0.2
	1.25		0.06	5.7 ± 0.1	17.9 ± 0.3
	1.25		0.06	5.6 ± 0.0	18.0 ± 0.4
	0.5		0.03	5.4 ± 0.1	11.6 ± 0.1
	2		0.03	5.5 ± 0.1	17.4 ± 0.2
	0.5		0.1	5.2 ± 0.1	15.5 ± 0.2
	2		0.1	5.5 ± 0.1	17.9 ± 0.4
	0.19		0.06	5.1 ± 0.0	13.4 ± 0.4
Chitosan	2.31	Oregano	0.06	5.6 ± 0.1	18.5 ± 0.3
	1.25		0.01	5.4 ± 0.1	16.6 ± 0.2
	1.25		0.12	5.4 ± 0.1	14.9 ± 0.3
	1.25		0.06	5.4 ± 0.0	14.7 ± 0.2
	1.25		0.06	5.5 ± 0.1	14.9 ± 0.3
	1.25		0.06	5.4 ± 0.0	14.7 ± 0.1
